# Vertebrate centromere architecture: from chromatin threads to functional structures

**DOI:** 10.1007/s00412-024-00823-z

**Published:** 2024-06-10

**Authors:** Lorena Andrade Ruiz, Geert J. P. L. Kops, Carlos Sacristan

**Affiliations:** 1 Hubrecht Institute, Royal Netherlands Academy of Arts and Sciences, Utrecht, Netherlands; 2 University Medical Center Utrecht, Utrecht, Netherlands; 3 Oncode Institute, Utrecht, Netherlands

**Keywords:** Centromere, Kinetochore, CENP-A, Chromatin organization, Epigenetics

## Abstract

Centromeres are chromatin structures specialized in sister chromatid cohesion, kinetochore assembly, and microtubule attachment during chromosome segregation. The regional centromere of vertebrates consists of long regions of highly repetitive sequences occupied by the Histone H3 variant CENP-A, and which are flanked by pericentromeres. The three-dimensional organization of centromeric chromatin is paramount for its functionality and its ability to withstand spindle forces. Alongside CENP-A, key contributors to the folding of this structure include components of the Constitutive Centromere-Associated Network (CCAN), the protein CENP-B, and condensin and cohesin complexes. Despite its importance, the intricate architecture of the regional centromere of vertebrates remains largely unknown. Recent advancements in long-read sequencing, super-resolution and cryo-electron microscopy, and chromosome conformation capture techniques have significantly improved our understanding of this structure at various levels, from the linear arrangement of centromeric sequences and their epigenetic landscape to their higher-order compaction. In this review, we discuss the latest insights on centromere organization and place them in the context of recent findings describing a bipartite higher-order organization of the centromere.

## Introduction

Centromeres are regions of specialized chromatin that form the primary constriction of the mitotic chromosome and have crucial functions for cell division (Flemming 1879; Fukagawa and Earnshaw 2014; McKinley and Cheeseman 2016; Schalch and Steiner 2017). These loci are defined structurally and functionally by the deposition of a centromere-specific Histone 3 (H3) named Centromeric protein A (CENP-A) (Earnshaw and Rothfield 1985; Kingwell and Rattner 1987; Palmer et al. 1987, 1991). In vertebrates, CENP-A spans several hundred kilobases, forming the core centromere (Altemose et al. 2022a; Logsdon et al. 2024). CENP-A’s main function is to direct the assembly of the kinetochore, the structure responsible for connecting centromeres to spindle microtubules during mitosis (Fukagawa and Earnshaw 2014). The regions flanking the core are known as pericentromeres and have critical functions such as ensuring sister chromatid cohesion, which is essential for generating tension and stabilizing kinetochore-microtubule interactions (Ng et al. 2009; Tanaka et al. 1999, 2000).

**Fig. 1:**
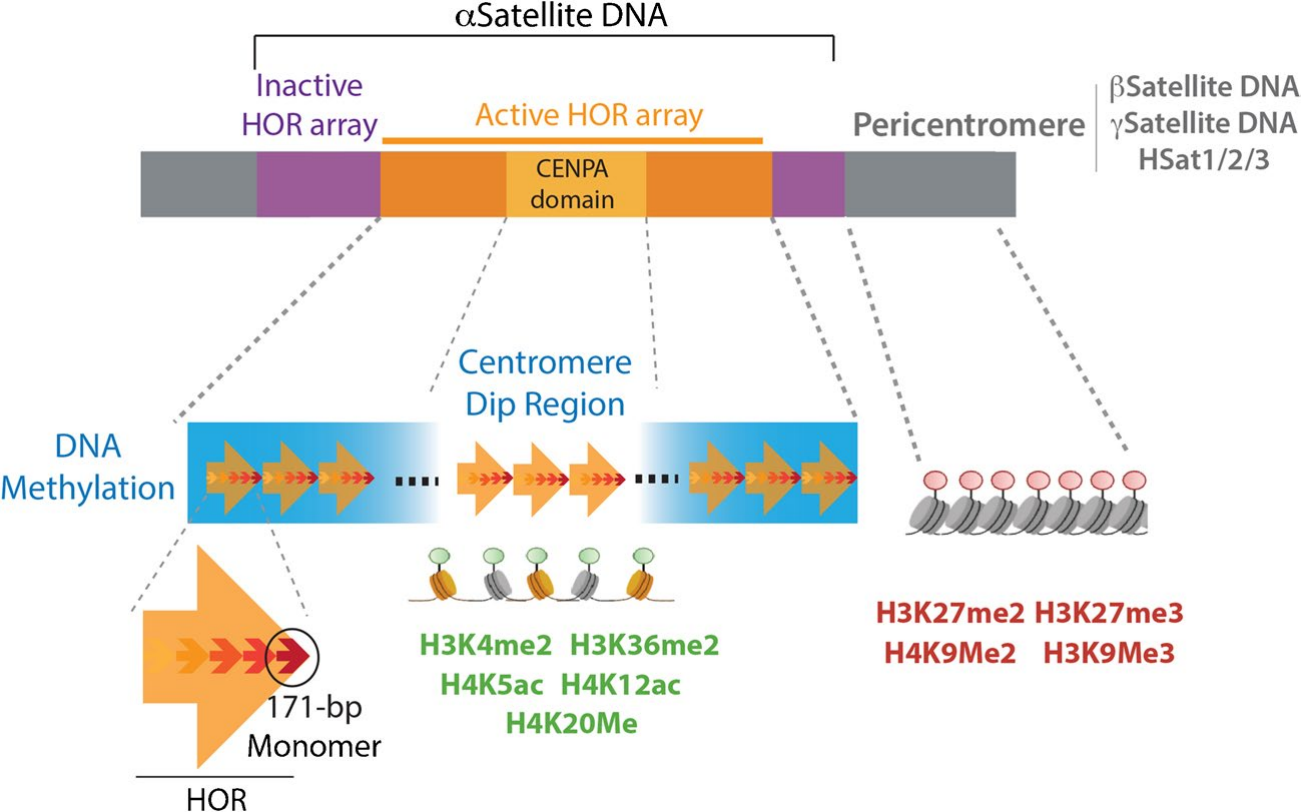
Genetic and epigenetic features of the centromere. Schematic of the genetic and epigenetic elements that compose the core centromere and pericentromere, indicating the inactive and active HOR arrays, and the CENP-A-binding domain. αSat monomers are portrayed as smaller arrows within the HORs. The DNA methylation pattern of the active array is shown in blue, and the centromere dip region (CDR) is indicated. Euchromatic (green) and heterochromatic (red) epigenetic marks present at the core centromere and pericentromere are depicted as circles located on top of the CENP-A (yellow) and H3 (gray) nucleosomes

In humans, (peri)centromeres are largely comprised of highly repetitive sequences known as satellite sequences (Alexandrov et al. 1988; Rudd et al. 2003; Waye and Willard 1989; Willard and Waye 1987a). CENP-A is almost exclusively loaded within α-satellite (αSat) DNA consisting of AT-rich 171 bp-long monomers that are tandemly repeated in a head-to-tail fashion forming higher-order repeats (HORs) (Vafa and Sullivan 1997; Altemose et al. 2022a; Rudd and Willard 2004; Willard and Waye 1987b). HORs are chromosome-specific, and they differ in the type, order, and number of monomers (Altemose et al. 2022a; Logsdon et al. 2024; Willard and Waye 1987a). A subset of these monomers contains a 17-bp sequence called the “CENP-B Box”, a motif recognized by Centromeric Protein B (CENP-B) (Masumoto et al. 1989; Muro et al. 1992) which enhances the epigenetic robustness of the centromere. HORs are further arranged into highly homogeneous arrays that can span kilobase to megabase-long regions (Altemose et al. 2022a; Logsdon et al. 2024; Warburton and Willard 1990; Willard and Waye 1987b). Although several arrays may be present per centromere, only a subset of HORs within a single array, known as the active array, are occupied by CENP-A (Altemose et al. 2022a; Gershman et al. 2022; McNulty and Sullivan 2018). Notably, satellite sequences and CENP-B are nonetheless not essential for centromere identity: chromosome Y lacks CENP-B boxes (Earnshaw et al. 1987, 1989; Miga et al. 2014), and CENPA can occupy, experimentally or naturally, non-repetitive sequences and create a functional neocentromere (Debose-Scarlett and Sullivan 2021; Murillo-Pineda et al. 2021; Naughton and Gilbert 2020). In canonical centromeres, pericentromeres flanking HOR arrays are composed of more degenerated and variable sequences, including β and γ-satellite DNA and satellite DNA I, II, and III (Altemose et al. 2022a; Hoyt et al. 2022; Logsdon et al. 2024; Smurova and De Wulf 2018). Additionally, pericentromeres contain non-LTR autonomous retrotransposons, DNA transposons, and retroviral elements (Altemose et al. 2022a; Hoyt et al. 2022; Smurova and De Wulf 2018). The core centromere and the pericentromere also show distinct epigenetic signatures, with pericentromeres typically associated with heterochromatin while the core centromere shows traits related to open chromatin (Fig. 1) (Fukagawa and Earnshaw 2014; Smurova and De Wulf 2018). This special epigenetic landscape creates a unique chromatin configuration suitable for the recruitment of the Constitutive Centromere-Associated Network (CCAN), a complex that works as a link between centromeric chromatin and the microtubule-binding region of the kinetochore (Hara and Fukagawa 2017; Hori et al. 2008, 2013; McAinsh and Meraldi 2011; Musacchio and Desai 2017; Perpelescu and Fukagawa 2011).

The structural maintenance of chromosomes (SMC) complexes are also enriched in centromeric chromatin. In animals, cohesin, condensin I, and condensin II are the most prominent of these multi-protein complexes with ATPase activity that orchestrate the 3D organization of chromatin, and which have critical functions in genome regulation and chromosome segregation (Davidson and Peters 2021; Hoencamp and Rowland 2023; Uhlmann 2016). The three complexes have well-described roles in centromere maintenance and function: from mediating sister chromatid cohesion and chromosome biorientation for cohesin (Tanaka et al. 1999, 2000), to ensuring pericentromeric compliance and preserving core centromere integrity in response to spindle forces for the condensins (Gerlich et al. 2006; Oliveira et al. 2005; Ribeiro et al. 2009; Samoshkin et al. 2009).

In chromatin fibers, centromeric chromatin exhibits a distinctive "beads on a string" linear arrangement, featuring discrete clusters of CENP-A nucleosomes interspersed among canonical nucleosomes (Blower et al. 2002; Haaf and Ward 1994; Kyriacou and Heun 2018; Ribeiro et al. 2010; Sullivan and Karpen 2004; Vargiu et al. 2017; Zinkowski et al. 1991). This arrangement has spurred the proposal of various models explaining how the CENP-A nucleosomes could come together in 3D during mitosis, including looping, helicoidal, and sinusoidal architectures (Blower et al. 2002; Fukagawa and Earnshaw 2014; Ribeiro et al. 2010). However, the precise architecture of centromeric chromatin remains elusive. The aim of this review is to highlight the most relevant contributions to our current understanding of centromere folding mechanisms. Additionally, building upon our recent findings that describe centromeres as bipartite structures (Sacristan et al. 2024), we discuss a model that explores how these diverse mechanisms might collectively contribute to the intricate process of centromere folding.

## Genetic and epigenetic features of the human centromere

The highly repetitive complexity of centromeric sequences has posed a significant challenge to our understanding of centromere biology. With the recent publication of the first complete assemblies of human centromeres, a breakthrough has been made toward comprehending the organization of these unique genomic regions and their evolutionary dynamics (Altemose et al. 2022a; Gershman et al. 2022; Hoyt et al. 2022; Logsdon et al. 2024). Phylogenetic analyses of the new assemblies reveal that the HOR array containing the core centromere, known as the active array, is more conserved and repetitive than the flanking centromeric regions. This organization likely results from a layered expansion of αSat repeats, where new repeats periodically emerge within the CENP-A region through a mechanism akin to tandem duplication (Altemose et al. 2022a). This has resulted in the progressive displacement of older repetitive sequences to the sides, which eventually have degenerated and diversified into the smaller, less repetitive, and more divergent satellite families (Altemose et al. 2022a; Shepelev et al. 2009).

The core and pericentromere exhibit distinct epigenetic signatures. The core is characterized by poised and activating marks such as H3K4me2, H3K36me2, H4K20me1, H4K5, and K12 acetylation, whereas pericentromeres feature a significant enrichment of constitutive heterochromatin histone modifications, such as H3K9me2/3 (Fig. 1) (Fukagawa and Earnshaw 2014; Gershman et al. 2022; Smurova and De Wulf 2018). Nanopore sequencing revealed that the active HOR array typically displays a higher DNA methylation content than the neighboring inactive HORs. This enrichment is locally interrupted at the so-called centromere dip region (CDR), which closely coincides with the site of CENP-A deposition (Fig. 1) (Altemose et al. 2022a; Gershman et al. 2022; Logsdon et al. 2021). Consistent with the euchromatic environment of core centromeres, RNA Pol II has been found associated with them (Chan et al. 2012; Perea-Resa and Blower 2018), and αSat transcripts are detected throughout the cell cycle (Hoyt et al. 2022). Centromere transcription facilitates CENP-A incorporation (Bobkov et al. 2018) and maintains the heterochromatic state of the pericentromere (Johnson et al. 2017), among several other functions (Perea-Resa and Blower 2018). An open chromatin state associated with RNA Pol II at the core and a compacted heterochromatic pericentromere have also been reported in neocentromeres (Murillo-Pineda et al. 2021; Naughton et al. 2022). Even though this epigenetic landscape is not universally present in all neocentromeres (Alonso et al. 2010; Nishimura et al. 2019), the fact that chicken neocentromeres lacking repressive marks have been found associated with H3K9Me3-dense regions in the nucleus (Nishimura et al. 2019) suggests an important interplay between epigenetic marks within the (peri)centromere to guarantee centromere identity and functions.

## Heterogeneity of human centromere structure

A surprising aspect arising from the first two assemblies of human centromeres (CHM1 and CHM13) is a remarkable heterogeneity between chromosomes and genomes in length, sequence, and position of the CENP-A domain (Altemose et al. 2022a; Logsdon et al. 2024). For instance, the length of active arrays ranges from 300 kb- 6.5 Mbs. Likewise, the extent of the CENP-A domain shows variability between genomes and centromeres, with the largest core (573 kb in Chr.1 of CHM13) being more than three times bigger than the smallest one (175 kb in Chr.9 of CHM13) (Altemose et al. 2022a; Logsdon et al. 2024). Notably, even smaller cores have been identified in human neocentromeres (Alonso et al. 2010; Murillo-Pineda et al. 2021; Naughton et al. 2022).

CENP-A in the centromere has been reported to be in excess (Bodor et al. 2014), potentially buffering the observed size differences between centromeres. Interestingly, a variability in CENP-A molecules has been found between centromeres and cell lines, ranging from ˜50–300 CENP-A nucleosomes per centromere (Bodor et al. 2014). Based on these numbers, a density of 1:25 CENP-A:H3 nucleosomes has been estimated (Bodor et al. 2014). These calculations, however, were based on previous estimations of 1 Mb centromere size (Sullivan et al. 2011). Considering the precise mapping of CENP-A in the new assemblies showing that the average core extends ~ 200 kb (Altemose et al. 2022a; Logsdon et al. 2024), the real density of CENP-A nucleosomes is likely to exceed these earlier estimates by several folds. Accordingly, Dimelo-Seq, a protein mapping technique compatible with long-read sequencing, estimates that CENP-A is present in around one out of four nucleosomes within chromosome X centromeres of HG002 cells (Altemose et al. 2022b).

Overall, the first complete assemblies of centromeric sequences have yielded crucial insights into the diverse nature of the linear organization of centromeres. It will be of interest to understand whether such diversity influences the final 3D organization of centromeric chromatin in mitosis and to elucidate how folding mechanisms of centromeric chromatin are regulated in different scenarios. Next, we will explore several mechanisms involved in the assembly of this unique architecture.

## CENP-A

CENP-A stands out as the most divergent member within the family of H3 histones (Ali-Ahmad and Sekulić, 2020; Sullivan et al. 1994; Tachiwana et al. 2012). Like other histones, CENP-A features a conserved histone fold domain (HFD), consisting of three α-helices connected by two short loops (L1 and L2). This domain mediates interactions with other CENP-A and histone H4 molecules, leading to the formation of a tetramer. Core centromeric chromatin purified from cells indicates that the predominant form in human cells is an octamer formed by the CENPA/H4 tetramer in complex with another H2A/H2B tetramer (Camahort et al. 2009; Hasson et al. 2013).

CENP-A and H3-containing nucleosomes show some key differences. In CENP-A, the L1 loop is positively charged and is more exposed than in a canonical nucleosome, facilitating interactions with centromeric factors (Ali-Ahmad and Sekulić, 2020; Tachiwana et al. 2011). Additionally, CENP-A nucleosomes only bind a fraction of the DNA that canonical nucleosomes do. This shortened wrapping is due to differences in CENP-A’s αN helix, which is shorter compared to histone H3 (Tachiwana et al. 2011). The length of the αN determines the ability of the nucleosome to stabilize the DNA at the entry and exit sites, resulting in highly flexible DNA ends in CENP-A nucleosomes (Roulland et al. 2016; Panchenko et al. 2011; Tachiwana et al. 2011).

It has been proposed that the high flexibility of the CENP-A nucleosome modulates the higher-order organization of chromatin. In vitro reconstituted arrays of CENP-A nucleosomes exhibit a more condensed configuration than canonical ones (Panchenko et al. 2011), while displaying higher local mobility compared to histone H3 (Takizawa et al. 2020, Nagpal et al. 2023). These higher dynamics might help create an open chromatin state, increasing the accessibility to centromeric factors (Takizawa et al. 2020, Nagpal et al. 2023). In cells, the higher flexibility of CENP-A ends prevents linker histone H1 from binding to the centromere. Mutant CENP-A nucleosomes capable of recruiting H1 cause the delocalization of kinetochore proteins, indicating that the flexible ends of the CENP-A nucleosome are essential for kinetochore assembly (Roulland et al. 2016).

**Fig. 2:**
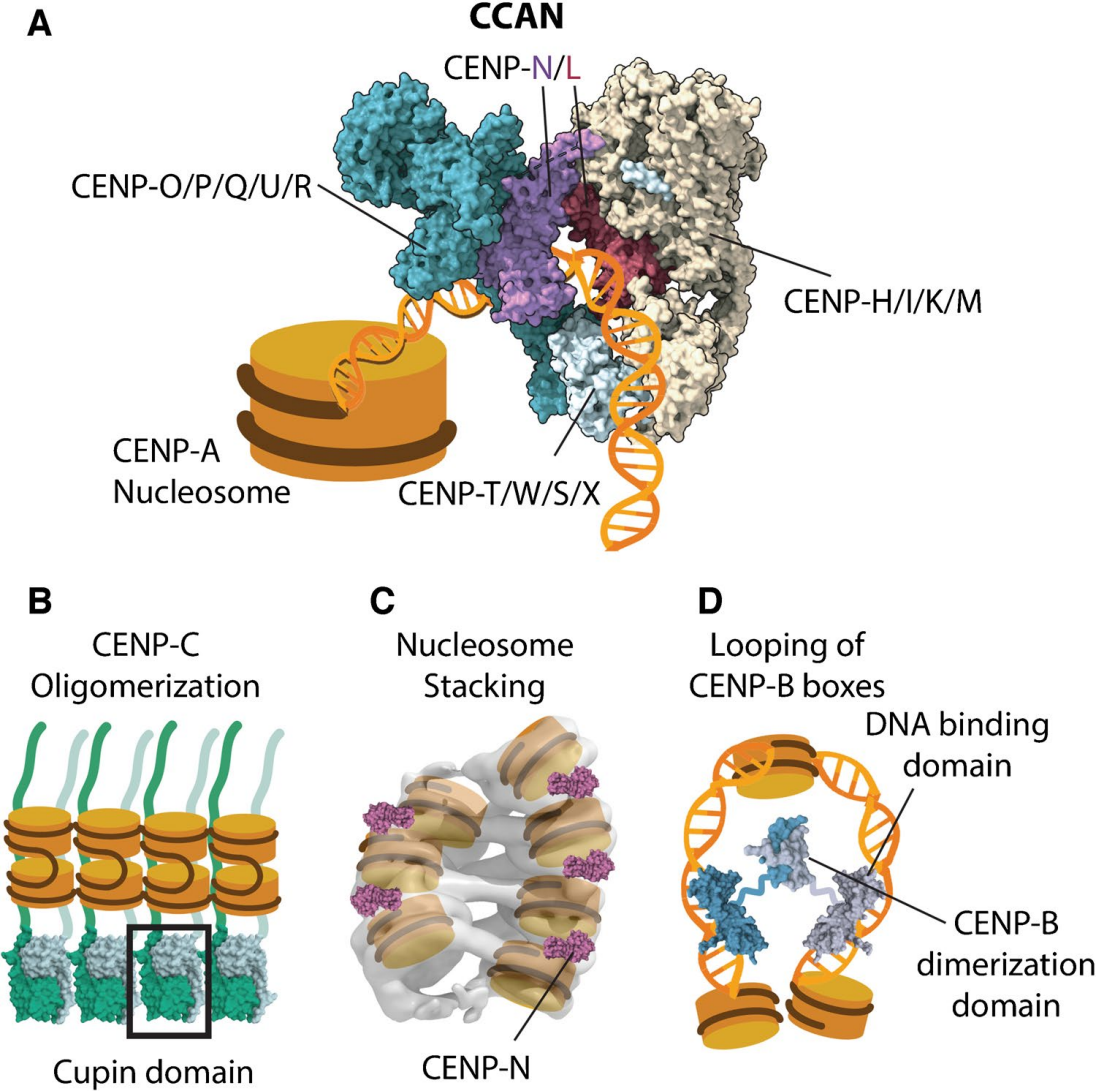
Centromeric organization by centromeric proteins. **a** Cartoon representing a CENP-A nucleosome bound to a CCAN complex (PDB 7QOO), not including CENP-C. **b** CENP-A nucleosomes organized by the oligomerization of CENP-C dimers (indicated in two shades of green). A homo-dimer of Cupin domains (PDB 7X85) is highlighted in the box. **c** CENP-A nucleosome-stacking driven by CENP-N (PDB 7U46). **d** α-satellite DNA (indicated in yellow) looping driven by CENP-B dimers (indicated in two shades of blue). The DNA binding (PDB 1HLV) and dimerization domains (PDB 1UFI) of CENP-B are indicated

Recent cryo-EM analyses of the human CCAN complex structure have uncovered how the extra nucleosomal DNA contributes to CCAN recruitment. The stable binding of CENP-A and the CCAN is mostly mediated by CENP-C (Pesenti et al. 2022; Yatskevich et al. 2022). This was a surprise since CENP-N had previously been postulated as a second major mediator of the nucleosome-CCAN interaction (Cao et al. 2018; Carroll et al. 2009, 2010; Chittori et al. 2018; Pentakota et al. 2017; Tian et al. 2018). With such a limited number of CENP-A-CCAN interactions, the stable recruitment of the CCAN is further supported by the linker DNA emerging from the CENP-A nucleosome (Pesenti et al. 2022; Yatskevich et al. 2022). This extra nucleosomal DNA is gripped by CENP-N/L, forming a tunnel through which the linker DNA threads. The CENP-HIKM and CENP-TWSX complexes close the tunnel and establish additional contact points with the DNA (Fig. 2a).

Taken together, the above studies suggest that the flexible ends of CENP-A nucleosomes aid in creating an unconstrained chromatin configuration, thereby potentially enhancing CCAN assembly by freeing up linker DNA.

## Centromeric proteins

While CENP-A nucleosomes can introduce significant alterations in chromatin arrangement, the ultimate higher-order organization of centromeric chromatin arises from a combination of mechanisms. Among them, centromere proteins CENP-C, CENP-N, and CENP-B are emerging as crucial factors influencing centromere compaction.

### CENP-C

Beyond its known functions mediating centromere-kinetochore associations (Hori et al. 2008; Klare et al. 2015; Przewloka et al. 2011; Saitoh et al. 1992; Screpanti et al. 2011; Sugimoto et al. 1994), CENP-C also influences centromeric chromatin organization. Overexpression of CENP-C in human cells induces chromatin clustering (Melters et al. 2019), while its depletion in chicken cells causes the unfolding of core centromeric chromatin (Vargiu et al. 2017) and a decrease in chromatin interactions (Hara et al. 2023). These interactions depend on a C-terminally located Cupin domain and its preceding 'pre-Cupin' region (Hara et al. 2023), necessary for CENP-C homodimerization and multimerization, respectively (Fig. 2b) (Chik et al. 2019; Cohen et al. 2008; Hara et al. 2023; Medina-Pritchard et al. 2020). In addition, CENP-C dimers are capable of binding two nucleosomes (Fig. 2b) (Walstein et al. 2021) providing another potential mechanism for nucleosome clustering.

Besides oligomerization, CENP-C promotes core centromeric compaction by reducing the intrinsic elasticity of the CENP-A nucleosome and by limiting the mobility of CENP-A nucleosomes (Melters et al. 2019, 2023).

### CENP-N

CENP-N is capable of establishing contacts with the L1-loop of CENP-A (Carroll et al. 2009, 2010; Chittori et al. 2018; Pentakota et al. 2017; Tian et al. 2018), however, this interaction is incompatible when CENP-N is integrated into the CCAN due to steric clashes (Pesenti et al. 2022; Yatskevich et al. 2022), While demonstrating a CCAN-independent role of CENP-N requires further investigation, recent work has shown that CENP-N can bind a second nucleosome in solution via electrostatic interactions with the DNA, facilitating the stacking of dinucleosomes and inducing a twisted double helix conformation in CENP-A arrays (Fig. 2c) (Zhou et al. 2022). Furthermore, expression of mutants defective in nucleosome-stacking properties resulted in significant decompaction of centromeric chromatin (Zhou et al. 2022).

### CENP-B

CENP-B targeting is dictated by the presence of CENP-B boxes within the centromeric sequences (Masumoto et al. 1989; Muro et al. 1992), which, in certain HORs, can be present in nearly every other αSat monomer. The affinity for the CENP-B box is diminished by CpG methylation (Y. Tanaka et al. 2005), which might explain the higher levels of CENP-B found within the CDR (Altemose et al. 2022a; Gershman et al. 2022).

CENP-B is dispensable for centromere formation and function (Earnshaw et al. 1989; Hudson et al. 1998; Kapoor et al. 1998; Masumoto et al. 1989; Perez-Castro et al. 1998). Nonetheless, CENP-B has been shown to promote centromere formation and enhance centromere fidelity (Fachinetti et al. 2015; Hoffmann et al. 2020). In addition, it modulates the centromere's epigenetic landscape by recruiting chromatin remodelers and histone chaperones (Okada et al. 2007; Otake et al. 2020).

CENP-B also develops structural functions. In highly homogenous arrays containing a CENP-B box every two αSat monomers, CENP-A is precisely positioned flanking both sides of the motif (Henikoff et al. 2015), suggesting that CENP-B might influence CENP-A phasing. Supporting this, CENP-B induces CENP-A nucleosome repositioning in in vitro reconstitutions (Chardon et al. 2022; Yoda et al. 1998). Through its N-terminal DNA-binding domain, CENP-B introduces kinks in DNA (Tanaka et al. 2001). In addition, by virtue of a C-terminal dimerization domain, CENP-B brings CENP-B boxes together creating loops in αSat sequences (Fig. 2d) (Chardon et al. 2022). Disruption of CENP-B dimerization results in impaired compaction and clustering of centromeres in interphase, and compromises centromere integrity in mitosis, suggesting that CENP-B-mediated looping contributes to the proper 3D organization of centromeric chromatin (Chardon et al. 2022).

## Condensin

Upon mitotic entry, chromosomes undergo intense condensation driven by the condensin complexes (Antonin and Neumann 2016; Batty and Gerlich 2019). Centromeres are particularly enriched in condensin (Gerlich et al. 2006; Oliveira et al. 2005; Ono et al. 2004; Ribeiro et al. 2009; Sacristan et al. 2024; Sutani et al. 2015; Walther et al. 2018), which plays a crucial role in ensuring centromere integrity. In chicken cells, condensin-depleted chromosomes show a decrease of 50% in the stiffness of pericentromeric chromatin when subjected to pulling forces (Ribeiro et al. 2009), and in humans, lack of condensin leads to severe centromere defects, often resulting in kinetochore fragmentation and merotelic attachments (Samoshkin et al. 2009).

Animals have two condensin complexes: condensin I and condensin II. Each of them has a differential distribution, abundance, and contribution to overall chromosome folding (Davidson and Peters 2021; Gibcus et al. 2018; Hoencamp and Rowland 2023; Uhlmann 2016; Walther et al. 2018). Both condensin complexes also show slightly different distributions within the centromere. In mitotic cells, condensin II shows a larger overlap with the core than condensin I (Ono et al. 2004). Conversely, in murine oocytes, condensin I is more prominently localized at the centromere compared to condensin II (Lee et al. 2011). Additionally, depletion of each condensin results in specific defects. Lack of condensin I, leads to an increased interkinetochore distance in mitosis (Gerlich et al. 2006; Uchida et al. 2009), which is consistent with impaired integrity of pericentromeric heterochromatin (Oliveira et al. 2005). In contrast, in mouse oocytes, the integrity of pericentromeric major satellite sequences crucially depends on condensin II levels (El Yakoubi and Akera 2023; Lee et al. 2011). This susceptibility creates a reproductive isolating between species with size differences in their major satellite sequences due to limiting condensin II levels in the oocytes of hybrids (El Yakoubi and Akera 2023).

## Cohesin

At mitotic onset, WAPL initiates cohesin removal from the chromosome arms, while at the centromere, Sororin, and Sgo1 counteract WAPL's action to protect cohesin at this location (Davidson and Peters 2021; Hoencamp and Rowland 2023; Uhlmann 2016). Safeguarding cohesin from WAPL is crucial to maintaining the tethering of sister chromatids, allowing tension development upon microtubule attachment (Tanaka et al. 2000).

The extent of centromeric cohesion distribution can be considered the physical boundary that functionally separates centromeres from the chromosome arm. The mapping of cohesin subunits in the latest full genome assemblies has revealed that cohesin specifically accumulates within the pericentromere, showing very poor enrichment at the active HOR, at least during interphase (Sen Gupta et al. 2023). While the precise mechanisms governing cohesin enrichment in these specific regions remain elusive, emerging evidence implicates transcription as a key factor. In *Saccharomyces cerevisiae*, cohesin loading predominantly occurs at the core centromere, facilitated by the Ctf19 complex (Hinshaw et al. 2017). Recent work indicates that cohesin subsequently migrates from the core towards the pericentromeric areas, where it becomes trapped by convergently transcribed genes (Paldi et al. 2020). Interestingly, in human cells, cohesin-rich pericentromeric regions also exhibit active genes alongside CTCF (CCCTC-binding factor) motifs (Sen Gupta et al. 2023; Xiao et al. 2015). CTCF protein binding of these motifs works as a barrier that impedes cohesin extrusion (Davidson and Peters 2021; Hoencamp and Rowland 2023). Similarly, the formation of a neocentromere in chromosome 3 was associated with heterochromatization of the pericentromere boundaries, coinciding with regions enriched in CTCF and flanked by genes transcribed toward the core (Naughton et al. 2022). Collectively, these observations underscore the role of transcription in establishing pericentromeric boundaries, where potentially, convergent transcription drives cohesin movement until it reaches CTCF-enriched sites, facilitating cohesin accumulation in these areas.

**Fig. 3:**
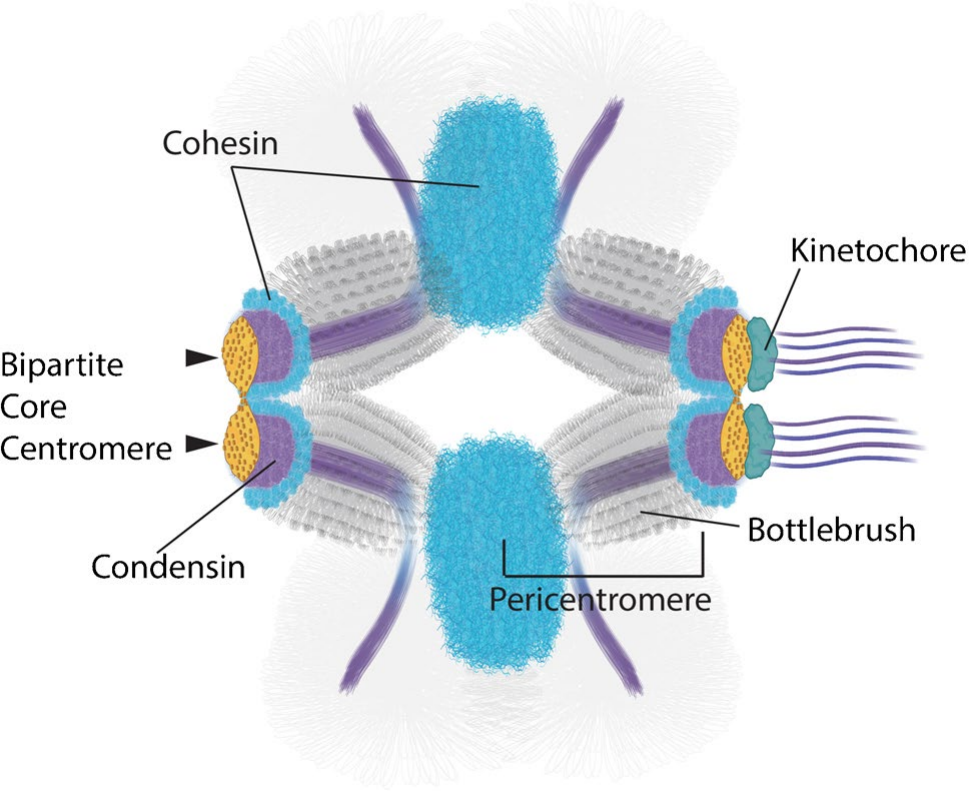
Bipartite higher-order organization of centromeric chromatin. Cartoon representing the centromeric region of a mitotic chromosome. Each subdomain of the bipartite centromere (yellow blobs) is associated with the flanking pericentromere, folded as a bottlebrush. Condensin (purple) extends along the central axis of the pericentromere and is enriched at each of the core centromere subdomains. The two pools of cohesin, one at the boundary of the pericentromere and one proximal to the core centromere are indicated in blue. A bipartite kinetochore (green) is bound by independent microtubule bundles

Super-resolution microscopy has revealed the existence of a second pool of cohesin in the proximity of the core centromere (Fig. 3) (Sacristan et al. 2024; Sen Gupta et al. 2023). Consistent with this, cohesin components in human cells have recently been found associated with CENP-U (Yan et al. 2024). In addition, two pools of Sgo1 mirroring the distribution pattern of cohesin have also been reported (Liu et al. 2013, 2015). As cohesin is not detected at HORs during interphase (Sen Gupta et al. 2023) it remains uncertain whether this secondary pool specifically accumulates at the core during mitosis or if levels remain below detection for ChIP-seq approaches (Sen Gupta et al. 2023). Cohesin can work in trans keeping sister chromatids entrapped, or in cis, extruding loops (Davidson and Peters 2021; Hoencamp and Rowland 2023). It will be crucial to investigate whether the two centromeric pools of cohesin reflect different topological entrapments of centromeric chromatin by cohesin.

## A bottlebrush organization of the pericentromere

The accumulation of cohesin in the distal pericentromere suggests the formation of a primary intramolecular loop, with the CENP-A at its apex (Sen Gupta et al. 2023; Yeh et al. 2008) On the other hand, the elongation of the pericentromere in the absence of condensin indicates that condensin is required for the compaction of the loop (Gerlich et al. 2006; Ribeiro et al. 2009; Stephens et al. 2011). Most of our understanding of how this compaction might occur has been primarily shaped by studies in *Saccharomyces cerevisiae* (Lawrimore and Bloom 2022), where SMC complexes adopt a distinct geometrical arrangement. Condensin extends along the axis that connects the sister kinetochores, while cohesin appears radially displaced from this axis (Stephens et al. 2011). The observed distribution of SMC complexes is compatible with a bottlebrush organization of the pericentromere, where the intramolecular loop is nested by condensin into arrays of loops, with condensin occupying the central axis of the bottlebrush (Lawrimore et al. 2016). In this model, the radial displacement of cohesin results from its role in crosslinking the loops of the bottlebrush which would help to support the mechanical properties of the spring. Given the conserved distribution of cohesin (Paldi et al. 2020; Sen Gupta et al. 2023) and the function of condensin in compacting the pericentromere (Lawrimore and Bloom 2019; Lee et al. 2011; Ribeiro et al. 2009), a bottlebrush organization would also align with the characteristics of the vertebrate pericentromere (Fig. 3).

## A bipartite core centromere

Zinkowski and Brinkley were the first to propose that the centromere consists of repetitive subunits that, upon chromosome condensation, coalesce into a compact higher-order organization suitable for kinetochore assembly (Zinkowski et al. 1991). Their hypothesis is supported by the observations that under different conditions, such as in MUGs (mitotic unreplicated genomes) and chromatin fibers, centromeres appeared fragmented into distinct substructures (Blower et al. 2002; Haaf and Ward 1994; Kyriacou and Heun 2018; Ribeiro et al. 2010; Sullivan and Karpen 2004; Vargiu et al. 2017; Zinkowski et al. 1991).

We recently observed that this higher-level structure of the core centromere comprises two main subdomains, with each subdomain tightly associated with its neighboring pericentromeric region (Fig. 3) (Sacristan et al. 2024). Of note, ring-like configurations of αSat sequences (Di Tommaso et al. 2023) and kinetochore components have been reported, which are particularly present in the absence of mature attachments (Wynne and Funabiki 2016). The observed rings might reflect a relaxed configuration of the bipartite centromere prior to compaction triggered by microtubule attachment. A bipartite centromere has crucial implications, particularly in the division of the kinetochore plate into two distinct subdomains that are functionally independent, as attested by the ability of each subdomain to bind a discrete bundle of microtubules (Sacristan et al. 2024). This unexpected behavior carries inherent risks, as subdomains can interact with microtubules originating from opposite spindle poles, resulting in merotelic attachments. The biorientation of subdomains from the same kinetochore could be a primary mechanism contributing to chromosomal instability as split kinetochores are frequently observed in lagging chromosomes (Cimini et al. 2001; Cojoc et al. 2016; Sacristan et al. 2024).

SMC complexes are key regulators of the bipartite centromere. The assembly of the two subdomains relies on condensin loading during the G2/M transition, and the lack of it results in highly disorganized centromeres (Sacristan et al. 2024; Samoshkin et al. 2009). On the other hand, chromatids depleted of cohesin exhibit subdomains severely separated and engaged in merotelic attachments, suggesting that cohesin plays a crucial role in keeping subdomains physically associated. Given the presence of the secondary pool of cohesin proximal to the core centromere (Sacristan et al. 2024; Sen Gupta et al. 2023; Yan et al. 2024) we hypothesize that this specific pool is responsible for tethering the subdomains.

## Outlook: from 'beads on a string' to a bipartite centromere

Overall, many fundamental questions about centromere architecture are still unanswered. Bipartition might represent one of several layers of complexity of centromere folding. Supporting this, centromere fibers prepared under low stringent conditions unfold into a discrete number of steps, usually ranging between 2 and 5 (Vargiu et al. 2017). This differs from fiber preparations using harsher conditions (Kyriacou and Heun 2018), or condensin depletions (Sacristan et al. 2024; Samoshkin et al. 2009), where the “beads on a string” organization of the centromere is unveiled. While the observed substructures may include linear arrays of CENP-A nucleosomes, it is also plausible that they constitute some basic form of nucleosome clustering. Notably, in immunoelectron microscopy images, discrete blocks of CENP-A appeared further organized into higher-order fibers of 30 nm (Marshall et al. 2008). Considering the crosslinking activities attributed to CENP-C and CENP-N, it is tempting to speculate that they play a role in orchestrating the assembly of basic blocks of CENP-A, which are then further arranged by condensin into two subdomains. Nonetheless, the potential contribution of CCAN components to the bipartite configuration cannot be disregarded. Besides the proposed mechanisms, other factors, such as the topoisomerase IIA (Nielsen et al. 2020; Spence et al. 2007), might be at play.

Given the distinct distributions of both condensin complexes and cohesin, it is important to assess their specific contributions to core centromere folding and the potential bottlebrush organization of the vertebrate pericentromere. In addition, despite significant variability in the length of active HORs (Altemose et al. 2022a; Logsdon et al. 2024), interkinetochore distances remain consistently uniform across chromosomes suggesting that variations in cohesion distribution or the extent of chromatin condensation might be necessary to accommodate the heterogeneity of centromeric sequences (Sen Gupta et al. 2023). Therefore, understanding how the SMC complexes accumulate at their specific locations remains fundamental to explaining the folding characteristics and functioning of the centromere. The unique epigenetic signature, accessibility, and transcriptional activity of centromeric chromatin could be major determinants of the distribution of the SMC complexes. Finally, aberrant centromeric structures have been associated with cancer and infertility (Barra and Fachinetti 2018; Lagirand-Cantaloube et al. 2017; Zielinska et al. 2019). Identifying the mechanisms disrupting centromere structure will be thus paramount in unraveling the origins of chromosomal instability.

More than 140 years since Fleming first described the centromere (Flemming 1879), it is remarkable that we have only just begun to scratch the surface of the intricately complex nature of centromeric chromatin architecture. The recent publication of the full centromere assemblies and the continuous development of 3D-genome analyses and super-resolution techniques open exciting possibilities to dissect and work towards the understanding of this elusive structure.

## Data Availability

No datasets were generated or analysed during the current study.
